# Assessing influences of climate change on highland barley productivity in the Qinghai-Tibet Plateau during 1978–2017

**DOI:** 10.1038/s41598-022-11711-w

**Published:** 2022-05-10

**Authors:** Zemin Zhang, Changhe Lu

**Affiliations:** 1 Key Laboratory of Land Surface Pattern and Simulation, Institute of Geographic Sciences and Natural Resources Research, Chinese Academy of Sciences, Beijing, 100101 China; 2 College of Resources and Environment, University of Chinese Academy of Sciences, Beijing, 100049 China

**Keywords:** Climate change, Climate-change impacts, Sustainability

## Abstract

Grain production is becoming increasingly vulnerable to climate change globally. Highland barley (HB) is the most important cereal crop in the Qinghai-Tibet Plateau (QTP), so assessing HB productivity and its response to climate change could help to understand the capacity of grain production and food security. This study simulated the potential yield of HB annually at 72 meteorological stations for 1978–2017 using the WOFOST model, and then analyzed the spatiotemporal changes of HB potential yield and climatic factors in the growing season. Further, the influence of climate change on HB potential yield was explored in different temperature zones (TZ). Results indicate that the annual average of HB potential yield ranged from 3.5 to 8.1 t/ha in the QTP, and it was averaged at 6.5 t/ha in TZ-3, higher than other zones. From 1978 to 2017, HB potential yield for the whole QTP decreased slightly by 2.1 kg/ha per year, and its change rates were 23.9, 10.1, − 15.9, − 23.8 and − 16.7 kg/ha/year from TZ-1 to TZ-5 (*p* < 0.05), respectively. In all zones, average (Tave), maximum (Tmax) and minimum temperature (Tmin) showed a significantly warming trend (*p* < 0.01), and Tmin increased by 0.53, 0.45, 0.44, 0.40 and 0.69 °C per decade, higher than that of Tave and Tmax. However, temperature diurnal range (TDR) and radiation (RA) showed a downward trend, and their decrease rates were far higher in TZ-5 and TZ-3. In TZ-1, ΔTDR was the critical factor to the change in HB potential yield, which would increase by 420.30 kg/ha for 1 °C increase of ΔTDR (*p* < 0.01). From TZ-2 to TZ-5, ΔRA was the critical factor, but the influence amplitude in terms of the elastic coefficient, decreased from 4.08 to 0.99 (*p* < 0.01). In addition, other factors such as ΔTmax in TZ-3 and ΔTmin in TZ-4 and TZ-5 also had an important influence on the potential yield. To improve the HB productivity in the QTP, suitable varieties should be developed and introduced to adapt the climate warming in different temperature zones. In addition, efforts are needed to adjust the strategies of fertilizers and irrigation applications.

## Introduction

Climate change has attracted more and more attention in scientific research, and become a major concern as well in general public during recent decades. The 5th assessment report of the Intergovernmental Panel on Climate Change (IPCC) indicated that the global average temperature increased by 0.74 °C over the past century^1^. In China, the average temperature increased by 1.17 °C from 1980 to 2017, but the solar radiation showed a downward trend with a decrease rate of 19.5 MJ/m^2^ per decade during 1958–2017^2,3^. The Qinghai-Tibet Plateau (QTP) is rather sensitive to global climate change, and thus shows a more significant warming trend at a much larger rate than other regions during past decades^4,5^. During 1901–2016, the average temperature was increased by 0.37 °C per decade in the QTP, far higher than the average of 0.23 °C per decade in China^6,7^.

Climate change influences substantially ecological environment and many aspects of social life, and has become a great challenge to sustainable development for human beings^1^. Increasing evidences indicate that agricultural production, as an important field related to food security, is becoming increasingly vulnerable to climate change^8–11^. There is little doubt that, in association with increasing temperature, the length of crop growing duration has been shorten in the temperature regions, which adversely affects the accumulation of crop dry matter and yield^11,12^. In cold regions, however, climate warming can extend the growing season and improve the photosynthetic rate for cereal crops^13,14^. Therefore, analyzing the change trend of crop productivity under different thermal conditions in the context of climate change and its response to different climatic factors in the QTP can contribute to the development of region-oriented adaption measures to cope with the climate warming and to rationally utilize agricultural resources.

Highland barley (HB) is the most important cereal crop in the QTP and in 2014, its sown area and production accounted for 45.0% and 38.0% of the total, respectively^15–17^. Therefore, assessing its productivity could help to understand the capacity of regional grain production and food security^18^. Up to now, three studies addressed this issue^16,19,20^. The results from Zhao et al. showed that the simulated potential yield of HB was between 6.8 and 7.3 t/ha during 1965–2013 for the whole QTP, estimated with the Thornthwaite Memorial model, while the results from Zhao et al. (2020) ranged from 8.6 to 9.6 t/ha during 1961–2018, using the two models including Miami and Thornthwaite^19^. Another study from Gong et al. estimated that average HB potential yield at 7 representative stations using DSSAT-CERES-barley model was 5.7–11.3 t/ha^16,20^. These studies either focused on HB potential yield at few stations, or did not validate the simulation results, resulting in unknown accuracy^21^.

Regarding the methods of assessing crop potential yield, there are three categories as statistical model, photosynthetic efficiency model and mechanistic crop model^22–26^. Compared to statistical model and photosynthetic efficiency model, such as the Thornthwaite Memorial model, the mechanism model integrates physiological processes such as photosynthesis, respiration transpiration and dry matter distribution, and considers the effects of climate and soil properties, and thus has higher accuracy^22,23^. The WOFOST is a classical mechanism crop model, and can simulate daily crop physiological processes and has been used to quantitatively assess crop potential yield in many regions of the world^27–30^.

Due to alpine climate and high spatial variation in temperature regime induced by terrain influence in the QTP, the response and sensitivity of HB crop to climate change are quite different over spatial and from those in low altitude areas^12,31^. So, this study collected the available data at all meteorological stations in the QTP during 1978–2017, to assess the potential yield of HB, based on the WOFOST model as calibrated with published observation data at experimental stations.

This study has two major aims. One is to simulate the potential yield of HB crop in the main HB planting area using the calibrated WOFOST model, and the other one is to analyze the influences of climate change in different temperature zones (TZ). In concrete, this study firstly calibrated the HB crop parameters based on published experimental data, and then simulated HB potential yield annually at selected 72 stations during 1978–2017 using the daily weather data. Thirdly, the change trends of HB potential yield and climatic factors in the growing season were analyzed using Mann–Kendall and Sen’s slope methods. At fourth, the influence of climatic factors on HB potential yield was quantified in different temperature zones based on Pearson correlation and Stepwise Multiple Linear Regression (SMLR). Finally, implications were suggested.

## Materials and methods

### Study area

**Figure 1:**
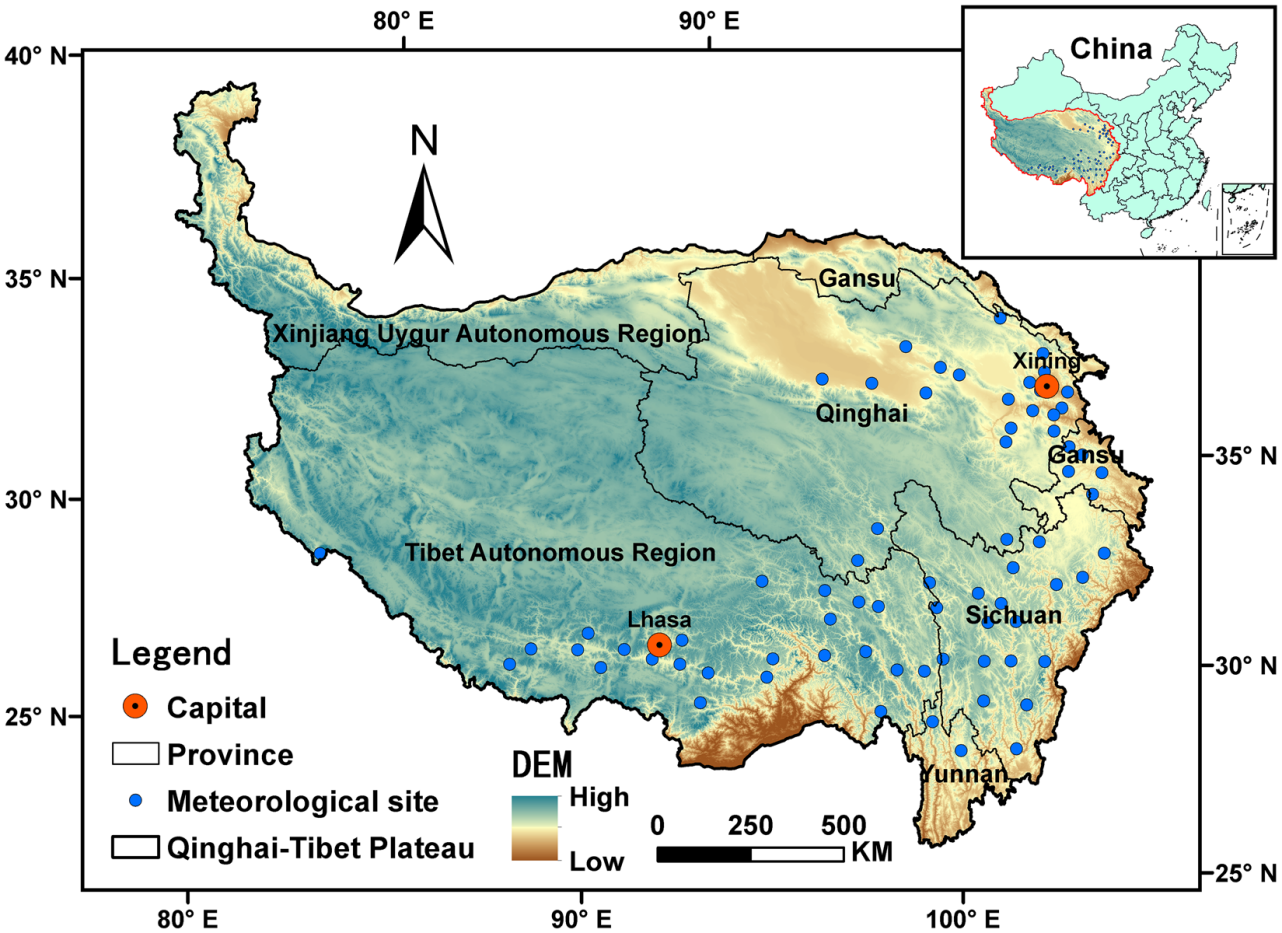
Boundary of the Qinghai-Tibet Plateau and location of 72 stations in the main sown region of highland barley. This map was generated using ArcMap 10.7 software ( © ESRI, https://desktop.arcgis.com).

The QTP (25° 20'–39° 30' N, 73° 20'–104° 20' E) has an average altitude over 4000 m and covers a total area of 2.68 M km^2^, involving the whole Tibet Autonomous Region and Qinghai Province, and parts of Sichuan, Gansu, Yunnan and Xinjiang provinces (Fig. 1). In the QTP, the annual average temperature ranges from − 1.5 to 16.6 °C, precipitation from 20.0 to 1658.0 mm, and solar radiation from 4235.5 to 8003.6 MJ/m^2^^32^, showing an obvious zonal and vertical differentiation with latitude and altitude^33^. The cultivated land is mainly distributed in the valleys of Brahmaputra River and its two tributaries, and the Yellow River and its tributary Huangshui River, under the altitude of 4300–4600 m a.s.l.^34,35^. The primary crops are highland barley, spring wheat, rapeseed, potatoes and peas, normally grown from early April to late September. The selected 72 meteorological stations in this study are mainly located at an altitude of 2000–4000 m in the planting areas of HB.

### Data sources and preprocessing

Daily meteorological data were sourced from the data center of resources and environment science of the Chinese Academy of Sciences (http://www.resdc.cn/Default.aspx), including average (Tave), maximum (Tmax) and minimum temperatures (Tmin), and average wind speed, sunshine hours, precipitation and relative humidity for 1978–2017. The soil data were obtained from the China dataset of soil hydraulic parameters with 1 km spatial resolution for land surface modeling^36^. Temperature diurnal range (TDR) and effective accumulated temperature (EAT) at each station were calculated directly based on daily data, and the solar radiation (Ra) and saturated vapor pressure were obtained using empirical functions of Angstrom-Prescott and Penman–Monteith equations (Eqs. 1, 2)^37,38^. Wind speed data are recorded at 10 m in the original weather file, and were converted to the value at 2 m height using Eq. (3)^39^. The original weather data were transformed to the specific format for WOFOST simulation using Python 3.6.7 programming. A total of 2960 available weather files were prepared for annual potential yield simulations.

1 $$Ra = Ra_{max} \cdot\left[ {{\text{a}} + {\text{b}}\left( \frac{n}{N} \right)} \right]$$

2 $$e_{a} = \frac{{RH_{mean} }}{100}\cdot\frac{{e^{0} \left( {T_{x} } \right) + e^{0} \left( {T_{n} } \right)}}{2}$$

3 $$V = V_{H} \cdot\left( {Z/Z_{H} } \right)^{\alpha }$$

where, *R*_*a*_ and *R*_*max*_ are the actual and maximum possible daily solar radiation (KJ/m^2^), and n and N are the actual and maximum daily sunshine hours (h), respectively. a and b are parameters related to atmosphere quality, setting to 0.27 and 0.55 in the QTP based on the FAO recommendation^40^. *Ea* is the actual vapor pressure (KPa), and *RHmean* is the average relative humidity (%). *Tx* and *Tn* are the maximum and minimum temperature, and *e*^*o*^*(Tx) and e*^*o*^*(Tn)* are the saturated vapor pressure (KPa) at maximum and minimum temperature. *V* is the wind speed at desired 2 m height; *V*_*H*_ is the wind speed at height *H* m; *Z* is the height to be revised, i.e., at 2 m; *Z*_*H*_ is the height of wind measurement (10 m); *α* is the wind speed variation index with height change, and the value is 0.16 in the QTP^39^.

### Estimation of trend and change rate

The trend was detected with the nonparametric Mann–Kendall test, a widely used approach to determine whether there is a significant trend in time series data^41,42^. The application of this method is mainly on the basis of two variables, *S* and *Z*, where *Z* is the normalized test statistic check value of the intermediate variable *S*. In the bilateral trend test, at a given confidence level α*,* if |*Z*|≥ Z_1–α/2_, it means a significant increasing or decreasing trend in the time series data; |*Z*|≤ Z_1–α/2_, means that no significant trend exists. The general forms are presented below:

4 $$S = \mathop \sum \limits_{i = 1}^{n - 1} \mathop \sum \limits_{{j = {\text{i}} + 1}}^{n} sgn\left( {X_{j} - X_{i} } \right)$$

5 $$Z = \left\{ {\begin{array}{*{20}c} {\left( {S - 1} \right)/{\upsigma }} \\ 0 \\ {\left( {S + 1} \right)/{\upsigma }} \\ \end{array} } \right.\begin{array}{*{20}c} { S > 0} \\ { S = 0} \\ { S < 0} \\ \end{array}$$

6 $$\sigma = \sqrt {n\left( {n - 1} \right)\left( {2n + 5} \right)/18}$$

where *X*_*i*_ and *X*_*j*_ are variables of maize yield in the *i*th and *j*th years; *n* is the length of the sequence data; *S* expresses the summation of sgn(*X*_*j*_–*X*_*i*_), which takes the value of − 1, 0 or 1 when (*X*_*j*_–*X*_*i*_) is less than, equal to or greater than 0, respectively; σ is standard deviation (SD).

The trend variability rate was estimated using the Sen’s slope method, a nonparametric estimation of the linear regression coefficients of the sequence data. It is usually used alongside the Mann–Kendall test to calculate the magnitude of change in a variable^43^. The general forms are expressed below:

7 $$Y\left( t \right) = SLOPE \cdot t + b$$

8 $$SLOPE_{i} = \frac{{Y_{j} - Y_{k} }}{j - k}$$

where *b* is the constant term, *i* denotes the county, *j* and *k* denote years (*j* > *k*), *SLOPE*_*i*_ is the Sen’s slope value of yield changes for county *i*, and *Y*_*j*_ and *Y*_*k*_ are the maize yield in the year* j* and *k*, respectively. If we let N be the time series length, *SLOPE* is presented as below:

9 $$SLOPE = \left\{ {\begin{array}{*{20}l} {SLOPE_{{\frac{{\left( {N + 1} \right)}}{2}}} } \hfill & {N\;is\;odd} \hfill \\ {\left( {SLOPE_{{\frac{N}{2}}} + SLOPE_{{\frac{{\left( {N + 2} \right)}}{2}}} } \right)/2} \hfill & {N\;is\;even} \hfill \\ \end{array} } \right.$$

In this study, the Excel template application MAESENS of the Mann–Kendall and Sen’s slope methods as developed by Salmi et al.^44^, were used to identify the change rates of HB potential yield and climatic factors and their significance during crop season for each station during 1978–2017.

### Potential yield simulation

In this study, WOFOST 7.1.7 was applied to simulate HB potential yield. WOFOST is a classical mechanism model, consists of several modules including photosynthesis, respiration, transpiration, nutrient cycling and dry matter distribution^45,46^, and performs well in simulating potential yield and crop phenology. The simulation process needs basic crop parameters, climatic data including maximum, minimum temperatures, radiation intensity, water vapor pressure, average wind speed and soil data such as soil texture, organic matter content and water conductivity^47–49^.

Before application of the WOFOST in the QTP, we calibrated the given crop parameters in the model. Firstly, a validation data set covering crop variety, sown date, emergence data, growing duration, experimental yield and trial years was built by collecting field experimental results in previously published papers (Table 1). Then, the required temperature sum (TSUM) for different varieties of HB for the periods of sowing-emergence, emergence-anthesis and anthesis-maturity were calculated and calibrated based on daily average temperature at corresponding meteorological stations according to the data set (Table 1). Finally, decision coefficient (*R*^*2*^), relative root mean square error (*RRMSE*) and percentage deviation coefficient (*PDC*) were adopted to evaluate the accuracy of simulated potential yield and growth duration with collected experimental data (Eqs. 10–14).

10 $$r = \frac{{\mathop \sum \nolimits_{i = 1}^{n} \left( {Y_{i}^{obs} - \overline{{Y_{i}^{obs} }} } \right)\left( {Y_{i}^{sim} - \overline{{Y_{i}^{sim} }} } \right)}}{{\sqrt {\mathop \sum \nolimits_{i = 1}^{n} \left( {Y_{i}^{obs} - \overline{{Y_{i}^{obs} }} } \right)^{2} \mathop \sum \nolimits_{i = 1}^{n} \left( {Y_{i}^{sim} - \overline{{Y_{i}^{sim} }} } \right)^{2} } }}$$

11 $$R^{2} = r^{2}$$

12 $$RMSE = \sqrt {\frac{{\mathop \sum \nolimits_{i = 1}^{n} \left( {Y_{i}^{obs} - Y_{i}^{sim} } \right)^{2} }}{n}}$$

13 $$RRMSE = \frac{RMSE}{{\overline{{Y_{i}^{obs} }} }}\cdot100{\text{ \% }}$$

14 $$PDC = \frac{{\mathop \sum \nolimits_{i = 1}^{n} (Y_{i}^{obs} - Y_{i}^{sim} )}}{{\mathop \sum \nolimits_{i = 1}^{n} Y_{i}^{obs} }}\cdot100\%$$

where, $Y_{i}^{obs}$ and $Y_{i}^{sim}$ indicate experimental and simulated HB yield at *i*th station; *n* is the number of stations.

**Table 1:** Data set for calibrating and validating WOFOST model parameters.

Station	Elevation (m)	EAT (°C·d)	Variety	Sown date	Emergence date	Growing duration (day)	Experimental yield (kg/ha)	Calibration data year	Validation data year	Reference
Dulan	3180	2192	Chaiqing-1	29th Apr	18th May	133	7460, 6045	2005, 2006	2006	^50^
Wulan	2950	2378		29th Apr	23rd May	133	6249, 7061	2005, 2006	2006	^50^
Datong	2450	2383		5th Apr	–	–	5700–6750	2009	2009	^51^
Gonghe	2930	2487		20th Mar.-10th Apr,	–	112–126	7923, 5250–6000	2010	2012	^52^
Golmud	2780	2866		27th Mar	26th Apr	102, 117	6795, 7125	2012, 2013	2013	^53^
Guinan	3120	2001		30th Mar.-24th Apr	20th Apr.-12th May	110–125	6027, 4384	2009–2013	2016–2017	^20^
Delingha	2980	2981		–	–	–	4500	2010	2011–2012	^54^
Menyuan	2860	1759		20th Mar.-15th Apr	10th Apr.-7rd May	125–140	5014, 4320	1980–2005, 2011–2015	2006–2015	^55^
Gannan	2936	2642		–	3rd May	133	6230	2012	2012	^56^
Lhasa	3658	3256	Zangqing-2000	4th May	13th Apr.-9th May	108–134	5172–7266	2008–2009	2011, 2014	^20,57^
Shigatse	3838	2779		20th Apr	3rd May	119–130	4800	2004–2008	2008–2015	^20,58^
Qamdo	3315	3030		–	10th Apr	110–116	4673	2013	2014	^59^
Pulan	3900	2078		–	7th May	125–135	3414	2018	2018	^60^
Lazi	4000	2776		20th Apr	–		5250–7420	2015–2016	2015–2016	^61^
Ganzi	3393	3394		–	10th Apr	108–130	6000	2013–2014	2013–2014	^62^
Linzhi	2991	3363		–	30rd Apr	112–115	5899	1994,2008	2000–2007	^20,58^
Shangri-La	3342	2511	Diqing-1	7th Apr	24th Apr	140	4064	2018	2018	^63^

– indicates a lack value of observation.

HB crop is generally sown when surface air temperature reaches 3 °C, and needs about 5–8 days for vernalization. Based on daily average temperature and phenological period at validation stations in Table 1, we obtained the TSUM parameters in the growing season. According to relevant literature and our field surveys in the QTP, Chaiqing-1 was generally planted in Qinghai and Gansu, Zangqing-2000 in Xizang and Sichaun and Diqing-1 in Yunnan, respectively, so we selected corresponding varieties to simulate HB potential yield in different regions^17^. Based on the normal sowing dates, and to be consistence for analysis of the response of HB potential yield to climate change, the sowing dates of Chaiqing-1, Zangqing-2000 and Diqing-1 was set on 10th, 20th and 30th April, respectively. All simulations are started from the sowing date and ended when the required TSUM is reached. The simulated results were analyzed and mapped using ArcGIS10.7.

### Influences of climate change on potential yield

Pearson’s correlation and stepwise multiple linear regression (SMLR) were applied to analyze the influence of climate change on HB potential yield. These two approaches were often used to analyze the relationship between climatic factors and crop potential yield and identify the critical factors by eliminating multicollinearity factors, respectively^8^. This study mainly analyzed the influences of six indices including Tave, Tmax, Tmin, effective accumulated temperature (EAT), temperature diural range (TDR) and solar radiation (RA) in the growing season. Firstly, their inter-annual changes i.e. ΔTave, ΔTmax, ΔTmin, ΔEAT, ΔTDR and ΔRA were obtained using first-difference time series (FDTS), which can eliminate the temporal trend of variables^20,64^. To reduce the influence of spatial climate variation, the 72 stations were classified into five TZ zones, i.e., < 2000, 2000–2500, 2500–3000, 3000–3500, > 3500 °C · d (Table 2), based on annual accumulated temperature above 0 °C. The analyses of Pearson’s correlation, SMLR and FDTS were conducted for each zone, using Stata 26.0, Origin Pro 8.0 and yield-climate panel data during 1978–2017.

**Table 2:** Meteorological stations in different effective accumulated temperature ranges of the Qinghai-Tibet Plateau.

Temperature zone	Temperature sum (°C·d)	Meteorological stations
TZ-1	< 2000	Qilian, Menyuan, Tongde, Dingri, Yushu, Luqu, Hezuo, Biru, Dingqing, Leiwuqi, Banma, Litang, Mangkang
TZ-2	2000–2500	Wulan, Dulan, Chaka, Huangyuan, Gonghe, Datong, Huangzhong, Hualong, Guinan, Xiahe, Pulan, Nanmulin, Jiangzi, Longzi, Nangqian, Aba, Luolong, Zuogong, Daocheng, Deqin, Rangtang
TZ-3	2500–3000	Delingha, Golmud, Nuomuhong, Tongren, Lazi, Shigatse, Nimu, Nozhugongka, Zhuoni, Diebu, Dege, Ganzi, Luhuo, Songpan, Xinlong, Kangding, Shangri-La
TZ-4	3000–3500	Guide, Ledu, Jianzha, Gongga, Lhasa, Zedang, Qamdo, Baiyu, Daofu, Maerkang, Heishui, Bomi, Linzhi, Milin, Jiulong
TZ-5	> 3500	Basu, Batang, Yajiang, Jiacha, Chayu, Muli

## Results

### Model calibration

**Figure 2:**
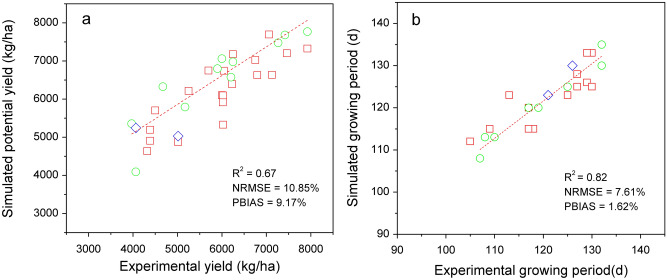
Comparison of simulated potential yield (**a**) and growing duration (**b**) of highland barley with observed data. Square, circle and diamond indicate the simulated and experimental results of Chaiqing-1, Zangqing-2000 and Diqing-1 varieties.

Based on the daily average temperature and experimental data, the required TSUMs for HB during the periods of sowing-emergence, emergence-anthesis and anthesis-maturity were determined (Table 3). The values of other crop parameters were not changed, i.e., the default parameters in the WOFOST crop database were used for simulations, and some of the main parameters were listed in Table 4. The simulation results were compared with the data in the validation dataset, and the results showed that *R*^*2*^ of potential yield and growing period were 0.67 and 0.82, *NRMSE* values were 10.85% and 7.61%, and *PDC* values were 9.17% and 1.62%, respectively (Fig. 2).

**Table 3:** The accumulated temperature required for each growth duration of spring wheat in the Qinghai-Tibet Plateau.

Variety	TSUM parameter in different growth periods (°C day)			
	Sowing-emergence	Emergence-anthesis	Anthesis-maturity	Emergence-maturity
Chaiqing-1	100	650	850	1600
Zangqing-2000	100	700	950	1750
Diqing-1	100	800	1050	1950

**Table 4:** The crop parameters of WOFOST model in simulating potential yield of highland barley in the Qinghai-Tibet Plateau.

Variable	Meaning	Unit	Values
AMAXTB	Maximum leaf CO_2_ assimilation	kg CO_2_ hm^−2^ h^−1^	35.0
SPAN	Life span of leaves growing at 35 °C	day	25.0
RGRLAI	Maximum relative increase in LAI	ha ha^−1^ day^−1^	0.0075
PERDL	Maximum relative death rate of leaves due to water stress	kg kg^−1^ day^−1^	0.02
RML	Relative maintenance respiration rate of leaves	kg CH_2_O kg^−1^ day^−1^	0.03
RMO	Relative maintenance respiration rate of storage organs		0.01
RMR	Relative maintenance respiration rate of roots		0.01
RMS	Relative maintenance respiration rate of stems		0.015
Q10	Relative change in respiration rate per 10 °C temperature change	kg hm^−2^	2.0

### HB potential yield and its change rate

**Figure 3:**
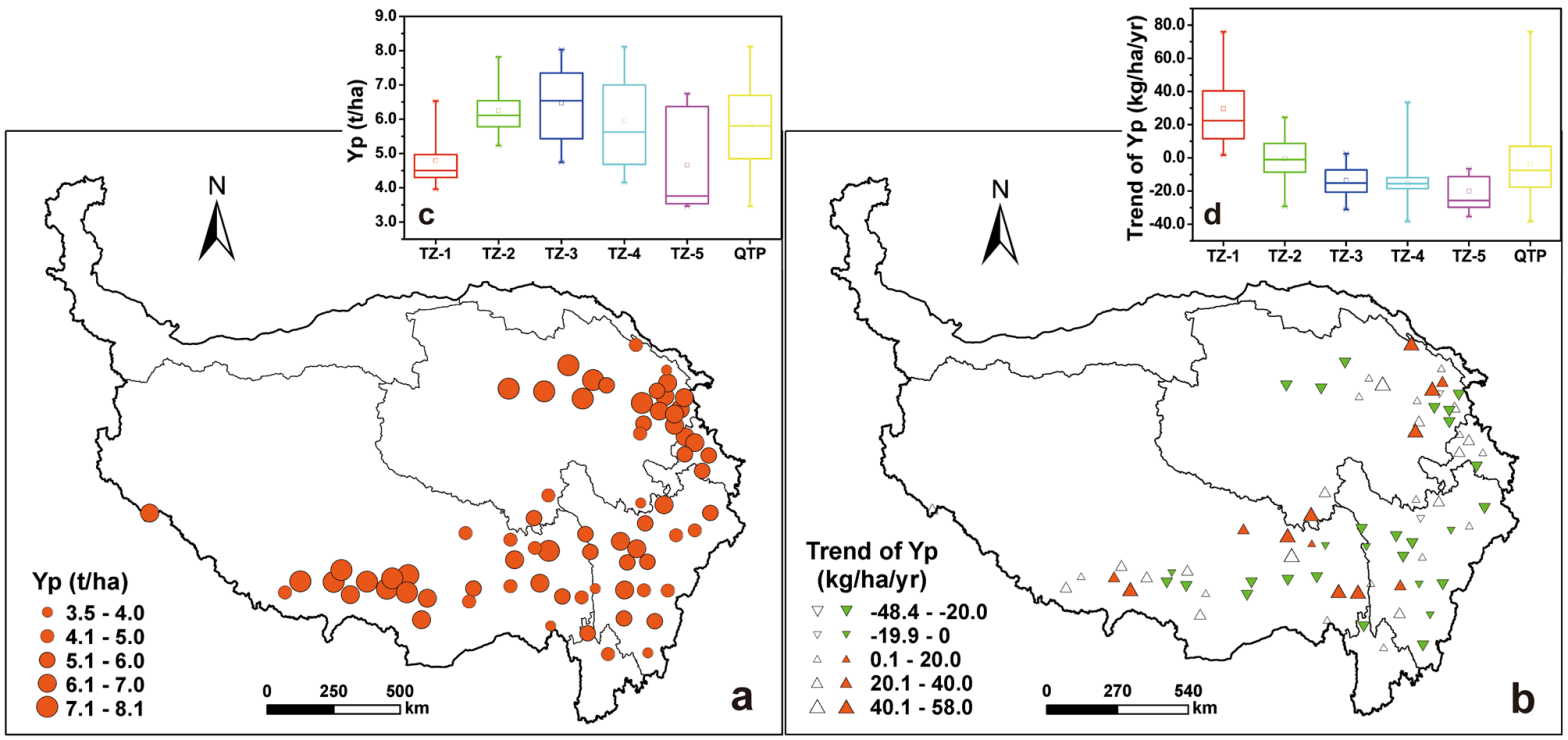
The annual average (**a**) and change trend (**b**) of HB potential yield in the Qinghai-Tibet Plateau during 1978–2017. Top-right box plots showed annual average (**c**) and change rate (**d**) of HB potential yield in different zones, respectively. Red and green triangles indicate significantly upward and downward trends (*p* < 0.05), and hollow triangle indicates insignificant trend. The size of triangle is proportional to change magnitude. Maps were generated using ArcMap 10.7 software ( © ESRI, https:// desktop.arcgis.com).

During 1978–2017, the annual average potential yield of HB ranged from 3.5 to 8.1 t/ha at 72 stations in the QTP. It was more than 6.0 t/ha at 34 stations, mainly concentrated in the southern Tibet and northern Qinghai. At 20 stations scattered in eastern Tibet and Sichuan, the potential yield was below 5.0 t/ha. At other 18 stations scattered in the whole QTP, HB potential yield was between 5.1 and 6.0 t/ha (Fig. 3a).

From 1978 to 2017, HB potential yield decreased slightly with a rate of 2.1 kg/ha/year in the whole QTP. At station level, the potential yield increased and decreased at 29 and 43 stations, and showed significant increasing and decreasing trends at 13 and 28 stations (*p* < 0.05), respectively. The change rate was between − 48.4 and 58.0 kg/ha/year at all stations, and was over 20.0 kg/ha/year at 37 stations scattered in Tibet and Qinghai (Fig. 3b). Ta other 35 stations, the change rate was relatively low, below 20 kg/ha/year. With the increase of temperature sum, the annual average of HB potential yield during the study period firstly increased and then decreased. In TZ-3, the potential yield was between 4.7 and 8.0 t/ha, averaged at 6.5 t/ha, higher than other zones (Fig. 3c). In TZ-1 and TZ-2, the mean potential yield was in an increasing trend, at a rate of 23.9 and 10.1 kg/ha/year, but in TZ-3 to Tz-5, it was decreased, averaged − 15.9, − 23.8 and − 16.7 kg/ha/year, respectively (Fig. 3d). At all stations in TZ-1 and 14 stations in TZ-2, HB potential yield had a positive change rate, while at 45 stations in other zones, it showed a decrease trend.

### Climate change in the growing season

**Figure 4:**
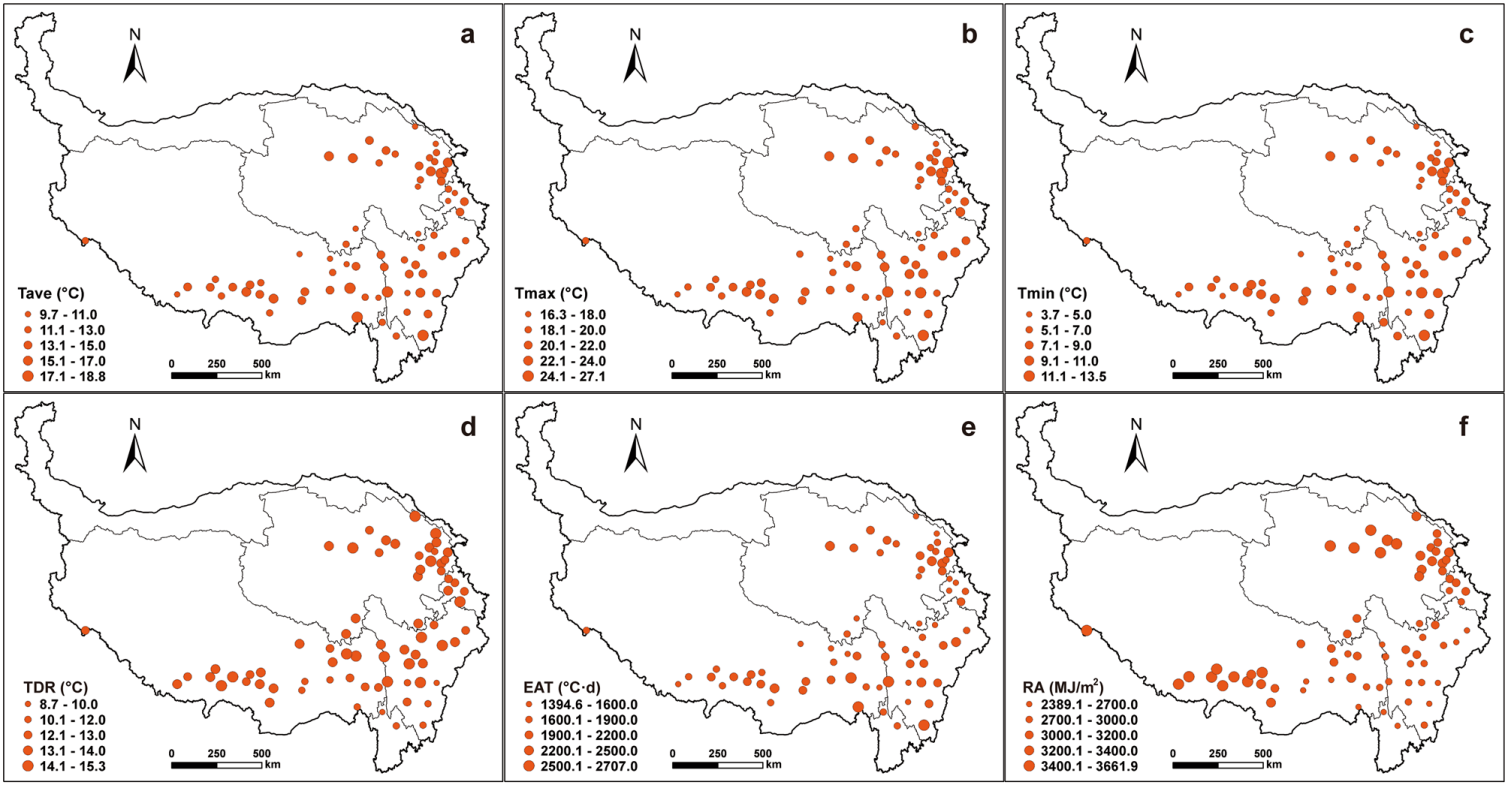
Long-term annual averages of Tave (**a**), Tmax (**b**), Tmin (**c**), TDR (**d**), EAT (**e**) and RA (**f**) in the growing season at 72 stations in the Qinghai-Tibet Plateau from 1978 to 2017. The size of circle is proportional to the values. Maps were generated using ArcMap 10.7 software ( © ESRI, https://desktop.arcgis.com).

In the QTP, the annual averages of Tave, Tmax, Tmin and TDR in the growing season during 1978–2017 ranged between 9.7–18.8, 16.3–27.1, 3.7–13.5 and 8.7–15.3 °C, with median values of 13.0, 20.1, 7.4 and 13.2 °C, respectively (Fig. 4a–f). EAT and RA were 1394.5–2707.0 °C day and 2389.1–3661.9 MJ/m^2^, respectively. The values of TDR and RA in southern regions were much lower than other parts.

**Figure 5:**
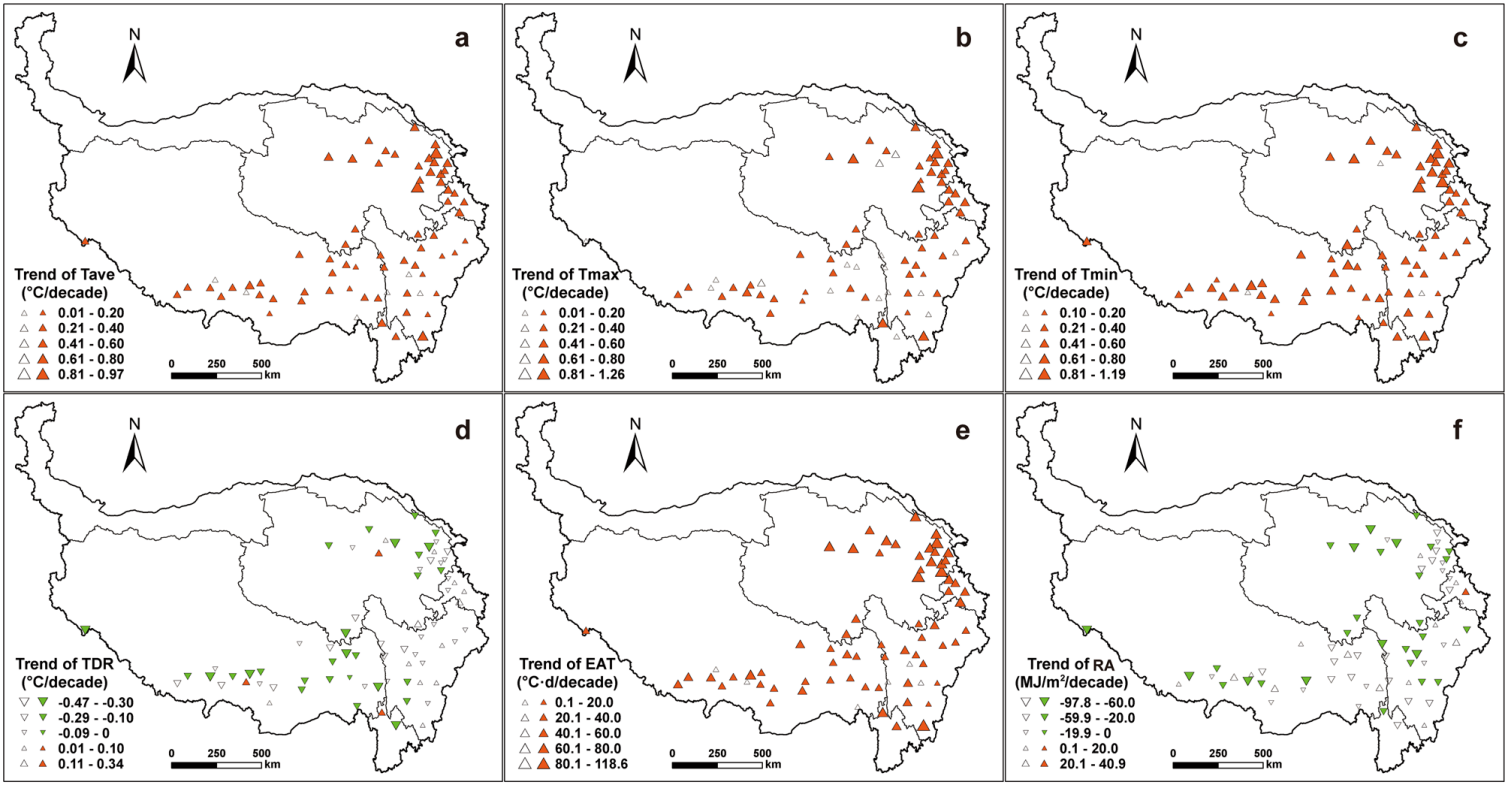
Change trends of Tave (**a**), Tmax (**b**), Tmin (**c**), TDR (**d**), EAT (**e**) and RA (**f**) in the growing season at 72 stations in the Qinghai-Tibet Plateau from 1978 to 2017. Red and green triangles indicate significantly upward and downward trends (*p* < 0.05), and the hollow triangle indicates insignificant trends. The size of triangle is proportional to change magnitude. Maps were generated using ArcMap 10.7 software ( © ESRI, https:// desktop.arcgis.com).

From 1978 to 2017, Tave, Tmax and Tmin increased at all stations, of which 66, 54 and 69 stations showed a significantly increase trend (*p* < 0.05), respectively. Their increase rates ranged 0.01–0.97, 0.01–1.26 and 0.10–1.19 °C per decade, averaged at 0.32, 0.33 and 0.47 °C per decade for the QTB, respectively. Spatially, Tave and Tmax increase rates exceeded 0.40 °C per decade at 17 and 19 stations, mainly concentrated in eastern Qinghai. At 46 stations located in the eastern Qinghai and southern Sichuan and Tibet, the change rate of Tmin was above 0.40 °C per decade, larger than other regions (Fig. 5a–c). Regarding TDR, 13 station showed an increasing trend, while 59 stations showed a decreasing and 27 stations a significantly decreasing trend (*p* < 0.05). The change rate of TDR was between -0.47 and 0.34 °C per decade at all stations, and at 17 stations mainly distributed in eastern Qinghai and Tibet, the decrease rate was below − 0.10 °C per decade, while at 5 stations scattered in the QTP, the increase rate was above 0.10 °C per decade. At other 40 regions, TDR showed a slight change trend. EAT showed significantly increasing trend at 66 stations (*p* < 0.05), and its change rate was above 40 °C day per decade at 38 stations. The trend and change rate of EAT were similar to that of Tave in spatial distribution. The change rate of RA was between − 97.8 and 40.9 MJ/m^2^ per decade at all stations and 79.2% of stations showed decrease trend. Of 57 stations with decrease rates, 27 stations located in southern Tibet, western Sichuan and Qinghai was identified with a significant trend (*p* < 0.05), and at 9 stations, the decrease rate was below 60.0 MJ/m^2^ per decade, far larger than other stations. (Fig. 5d–f).

**Figure 6:**
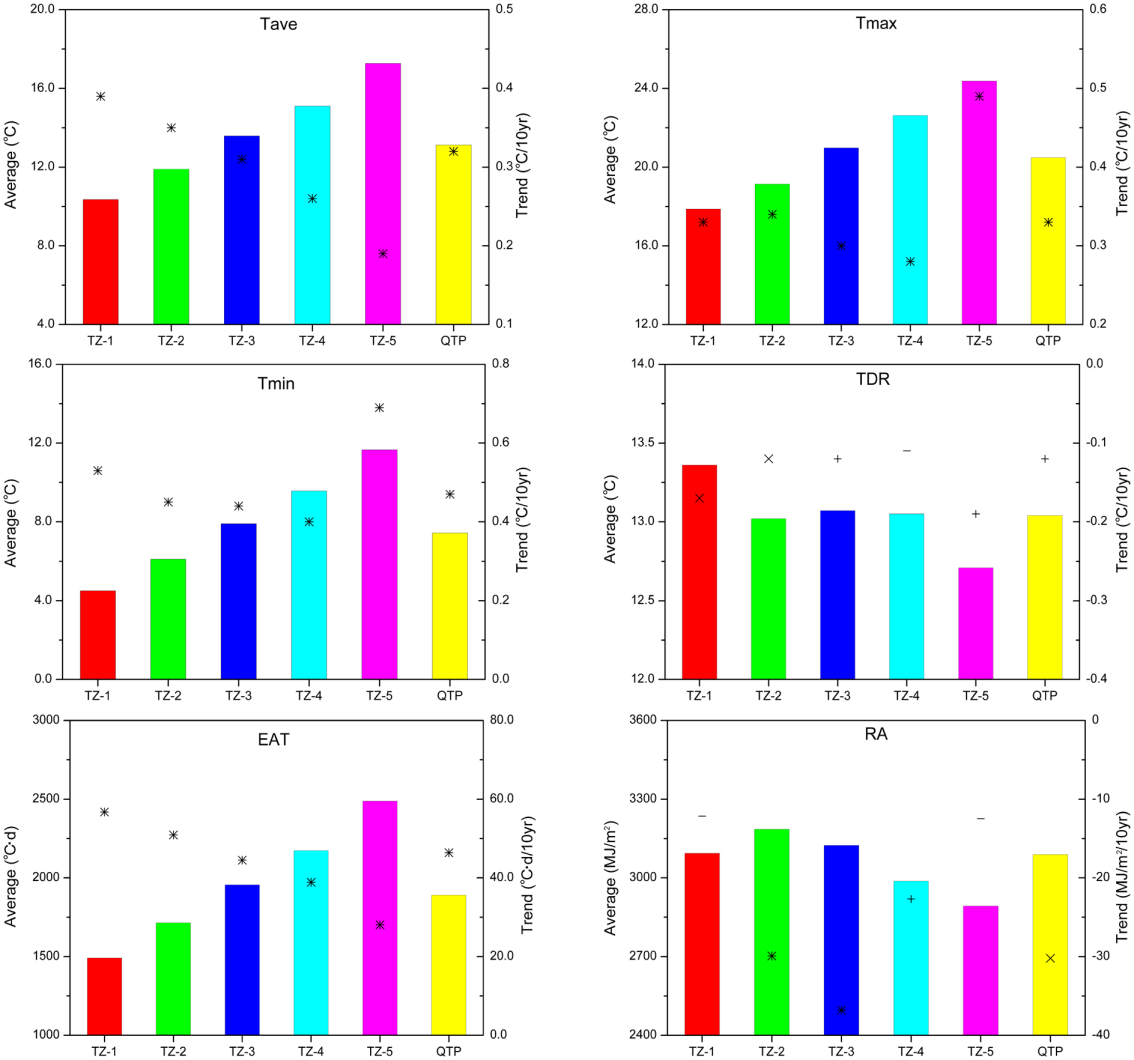
Annual averages (columns) and change trends (scatters) of Tave, Tmax, Tmin, TDR, EAT and RA in the growing season in different zones and the QTP. + , × and * indicate significant at 0.1, 0.05 and 0.01 levels, and − indicates insignificant change, respectively.

Further analysis to change rate of climatic factors in 5 temperature zones indicated that, Tave, Tmax, Tmin and EAT showed significantly upward trend in all EAT zones (*p* < 0.01), and their increase rates decreased with the rising EAT (Fig. 6). In addition, the increase rates of Tmin were 0.53, 0.45, 0.44, 0.40 and 0.69 °C per decade from TZ-1 to TZ-5, respectively, higher than that of Tave and Tmax. In all zones, TDR and RA showed decreasing trends, and the decrease rate of TDR first decreased and then increased with the rising temperature sum, while that of RA first increased and then deceased.

### Influences of climate change on HB potential yield

**Figure 7:**
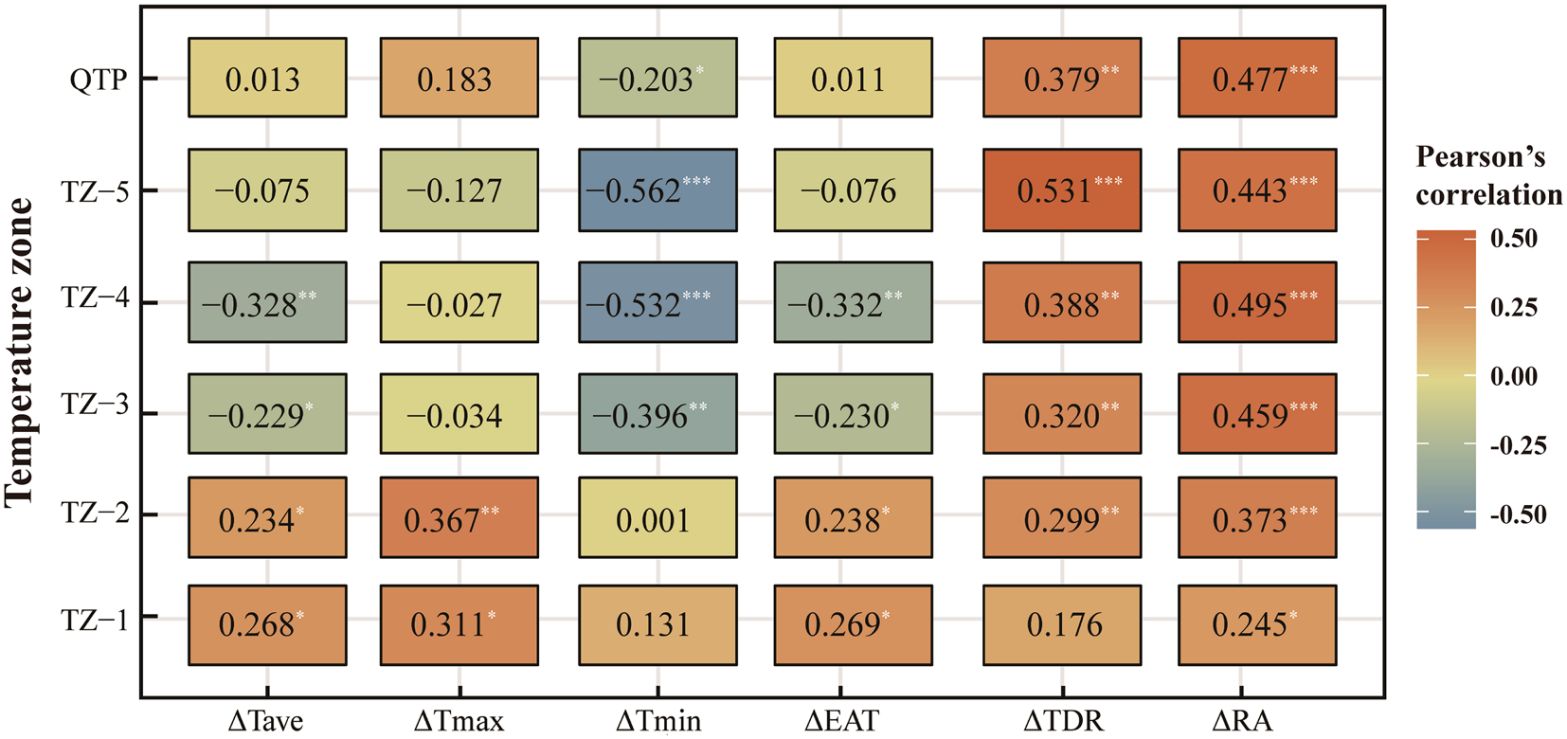
Pearson’s correlation coefficients between highland barley potential yield and climatic factors in different effective accumulated temperature zones and the Qinghai-Tibet Plateau. ΔTave, ΔTmax, ΔTmin, ΔEAT, ΔTDR and ΔRA represent inter-annual changes of average, maximum, minimum temperatures, effective accumulated temperature, temperature diurnal range and solar radiation in the growing season, respectively. *, **and *** indicate significant at 0.1, 0.05 and 0.01 levels, respectively.

For the whole QTP, all climatic factors had a significantly positive influence on ΔYp except for ΔTmin (*p* < 0.05), and the correlation coefficients of ΔRA and ΔTDR with ΔYp were larger than that of other factors (Fig. 7). ΔTave, ΔTmax and ΔEAT showed a significantly positive relationship with ΔYp in TZ-1 and TZ-2 zones (*p* < 0.05). From TZ-3 to TZ-5, however, the changes of ΔTave, ΔTmin and ΔEAT showed a significantly negative correlation (*p < *0.05). For ΔTDR and ΔRA, a significantly positive correlation was identified with ΔYp (*p* < 0.05), and the influence of ΔRA was slightly larger than that of ΔTDR. Totally, ΔRA and ΔTDR were positively correlated with ΔYp and the positive effects increased from TZ-1 to TZ-5. However, the influence of ΔTave, ΔTmax, ΔTmin and ΔEAT were different, i.e., with the rising temperature sum, their influence on HB potential yield generally changed from positive to negative, and the influence magnitude first increased and then decreased (Fig. 7).

The SMLR regression results passed the measurement test with R^2^ of 0.38, 0.46, 0.58, 0.61, 0.69 and 0.48 (*p* < 0.01) in all zones and the QTP (Table 5). For the whole QTP, the change in solar radiation was a critical factor to HB potential yield change (*p* < 0.01). Quantitively, ΔYp would increase by 2.34 kg/ha for 1 MJ/m^2^ increase in ΔRa. In TZ-1, ΔTDR was the critical factor, and for 1 °C increase of ΔTDR, ΔYp would increase by 420.30 kg/ha. From TZ-2 to TZ-5, ΔRA was still one of the critical factors but its elastic coefficient decreased from 4.08 to 0.99. Furthermore, there were other critical factors in different EAT zones, such as ΔTmax in TZ-3 and ΔTmin in TZ-4 and TZ-5.

**Table 5:** Step multiple linear regression results based on HB potential yield and climatic factors in different effective accumulated temperature zones and the whole Qinghai-Tibet Plateau during 1978–2017.

Temperature zone	Stepwise multiple linear regression	F	Sig	R^2^	RMSE
TZ-1	ΔYp = 420.30·ΔTDR − 0.98	6.12	0.02	0.38	463.79
TZ-2	ΔYp = 4.08·ΔRA + 9.12	10.69	0.00	0.46	685.50
TZ-3	ΔYp = 3.55·ΔRA − 368.79·ΔTmax − 3.23	9.02	0.00	0.58	414.03
TZ-4	ΔYp = 1.36·ΔRA − 354.03·ΔTmin − 10.52	10.49	0.00	0.61	354.83
TZ-5	ΔYp = 0.99·ΔRA − 316.40·ΔTmin − 1.55	16.76	0.00	0.69	232.42
QTP	ΔYp = 2.34·ΔRA − 4.78	10.87	0.00	0.48	380.68

## Discussion

### Uncertainty of HB potential yield simulation

In this study, the crop parameters in the WOFOST model were calibrated based on experimental data sourced from published papers, so the potential yield obtained in this study mainly referred to the highest yield level, assuming that water and nutrients are adequately supplied, pests and weeds are controlled well, and farming techniques and management measures are in their best conditions^65^. The simulated potential yield has a good agreement with the observation data set, and thus the results can provide a relatively reliable estimation of the potential yield in the QTP.

Furthermore, the QTP covers a large area involving seven provinces, so the sown date of HB crop may vary considerably. However, due to the lack of phenological data at most meteorological stations, we assumed the sown dates of HB crop according to the validation data set and did not change during the study period, which might cause some uncertainties to the simulated results at some stations. Nonetheless, the verification results showed that the simulated potential yield was in a good agreement with the validation data. Furthermore, these assumptions could also improve the sensitivity of HB potential yield to climate change and be beneficial to distinguish the influences of climatic factors, and made the estimation results comparable at both temporal and spatial scales.

### Climate change and its influence on HB potential yield

Our results indicated that the average values of daily Tmax, Tmin and Tave in the growing season across the QTP, showing a significantly upward trend during 1978–2017 (*p* < 0.05), and their change amplitudes were larger than that in other regions of China^11,66^. However, RA was identified with significantly decreasing trend (*p* < 0.05), and its decrease rate was lower than that in the North China Plain and Northeastern China^67^. Furthermore, the increase rate of daily Tmin was higher than that of Tave and Tmax, and their increase rates decreased with the rising temperature sum, while the decrease rate of RA showed an increasing trend. Similar results were reported in previous studies^68,69^.

In this study, FDTS method was applied to calculate the inter-annual changes of potential yield and climatic factors, so as to avoid the influence from the long-term trends of potential yield and climate factors on their correlation relationships. This approach has been proven to be reliable for identifying the effect of climate change on crop yield in other studies^20,64^. In addition, to eliminate the serious multicollinearity between climatic factors, we also adopted the SMLR regression to identify the critical factors from all climatic factors.

According to our results, RA had a positive influence on potential yield of HB in all temperature sum zones, due to that RA affects the photosynthetic efficiency of crop leaves directly. The decrease in RA has negative influence on HB potential yield in the QTP, which was similar with other grain crops in the North China Plain and Northeast China^70–72^. However, some differences were found in different temperature zones regarding the influences of Tave and EAT on potential yield. The reason could be that temperature decides the crop production by influencing the rate of dry matter accumulation and controlling the length of growth duration simultaneously^9,49,73^. The growth of HB crop needs not only the suitable level to meet photosynthesis of its leaves, but also appropriate EAT to ensure the length of growth period. In zones with higher temperature sum, temperature increases would shorten crop growth period and thus lead to the decrease in potential yield. However, in zones with relatively low temperature sum, the EAT in the growing season cannot sufficiently meet the need of HB crop growth, and the lower temperature results in low photosynthetic efficiency of leaves, even brings frost harm to crop body^5^. Therefore, the warming in these temperature zones can improve the photosynthetic efficiency, and promote the increase of potential yield. These can also explain why the influence amplitude of EAT first decreased and then increased from TZ-1 to TZ-5. In addition, it should be noted that HB potential yield in TZ-5 decreased more slowly than that of TZ-4, mainly due to that RA had a larger influence on HB yield in TZ-4 than in TZ-5 (Fig. 7), and ΔRA showed significantly decreasing trend with 22.68 MJ/m^2^ per decade in TZ-4 (*p* < 0.05), far larger than 12.48 MJ/m^2^ per decade in TZ-5 (Fig. 6).

Our results also indicated that the influence amplitude of TDR increases with the rising temperature sum. It is known that daily Tmax and Tmin are distributed in the day and night, respectively. So, in low temperature sum regions, the increase of daily Tmax is beneficial to crop photosynthesis and thus the increase of dry matter accumulation in the daytime, while the increase of daily Tmin leads to the increase of dry matter consumption for respiration in the night^74^. Therefore, the decrease in daily TDR has a negative influence on HB potential yield in the QTP, due to that the increase in Tmax has more contribution to TDR than Tmin (Fig. 6). These results are consistent with other relevant studies^74,75^.

### Implication

In the QTP, agricultural infrastructure construction and field management levels are relatively low, and the application of pesticides and fertilizers are highly restricted in agricultural practice in some regions due to strict conservation of ecological environment. As a result, HB actual yield is still rather low and has a large yield gap. For instance, the average actual yield of HB crop during 2012–2017 was 2.2 t/ha in Qinghai province, and its average potential yield at 22 meteorological stations was 6.1 t/ha, with a yield gap of 3.9 t/ha. According to the IPCC report, the global average temperature will rise by 0.3–0.7 °C in 2035, and the change magnitude in the QTP will be far higher^1,76^. Therefore, in regions with lower temperature sum, HB potential yield will continue to increase in the future, leading to the increase of upper limit of altitude suitable for HB growth.

In filed surveys, we also found that the major restricting factor was the low ratio of irrigation guarantee, due to that available farmland is mainly terraced farmland on the hill slopes. For instance, only 35.1% of the arable land in Qinghai province was accessible for irrigation in 2017 (Statistical Yearbook). To improve crop yield, therefore, it is necessary not only to develop suitable varieties that can adapt the climate warming in different EAT zones, but also to adjust the strategies of fertilizers application and to improve irrigation guarantee rate.

## Conclusion

Based on the WOFOST model, Mann–Kendall and Sen’s SLOPE, SMLR and GIS spatial analysis, this study simulated HB potential yield and its change trend at 72 stations in the QTP during 1978–2017, and then explored its response to climate change in different EAT zones. Results showed that the annual average potential yield of HB ranged from 3.5 to 8.1 t/ha, and the potential yield was larger than 6.0 t/ha at 34 stations, which mainly located in southern Tibet and northern Qinghai. From 1978 to 2017, HB potential yield for the whole QTP decreased slightly by 2.1 kg/ha per year, and its change rates were 23.9, 10.1, − 15.9, − 23.8 and − 16.7 kg/ha/year from TZ-1 to TZ-5 (*p* < 0.05), respectively, meanwhile, Tmax, Tmin and Tave increased with change rates of 0.32, 0.33 and 0.47 °C per decade (*p* < 0.05). The change rates of Tmax, Tmin and Tave decreased with the increasing temperature sum, while that of RA and TDR mainly showed decreasing trends. RA and TDR were positively correlated with HB potential yield in all zones, while the influence of Tave, Tmax, Tmin and EAT changed from positive to negative with the increasing EAT, and the influence amplitude first increased and then decreased. To improve HB productivity, it is suggested that suitable varieties should be developed to adjust the climate warming in different temperature sum regions, and the strategies of fertilizers application and irrigation should be adjusted.

## Data Availability

Meteorological information used in this study are available at http://www.resdc.cn/Default.aspx.
